# Outcomes of adjuvant epithelial growth factor receptor tyrosine kinase inhibitors (EGFR-TKIs) treatment for EGFR-mutant non-small-cell lung cancer: a propensity-score analysis

**DOI:** 10.1038/s41598-017-11725-9

**Published:** 2017-09-14

**Authors:** Shufen Zhao, Ge Ma, Jing Guo, Aiping Ding, Shasha Wang, Guohong Yu, Lei Chen, Yonggang Yuan, Wenjing Xiao

**Affiliations:** 1 The Affiliated Hospital of Qingdao University, Qingdao, Shangdong Province China; 2 Yidu Center Hospital of Weifang, Weifang, Shandong Province China

## Abstract

Small molecule tyrosine kinase inhibitors (TKIs) have transformed the management of advanced non-small-cell lung cancer (NSCLC) harboring activating epithelial growth factor receptor (EGFR) mutations, while the efficacy of TKIs in the adjuvant setting remains unclear. We collected the data of 209 EGFR-mutant NSCLC patients receiving complete resection from 2010 to 2013. Study end points were disease-free survival (DFS) and overall survival (OS). Among the eligible patients, 41 (19.6%) received EGFR TKIs in the adjuvant treatment. The 3-year DFS of adjuvant EGFR TKIs treatment group (70.5%, 95% CI, 54.6–86.4%) was significantly superior that control group (50.2%, 95% CI, 40–60.4%; log-rank P = 0.014). TKIs treatment (HR, 0.51; 95% CI, 0.29–0.97; P = 0.04) was significantly associated with improved DFS in multivariate Cox analysis. No significant difference was observed in 3-year OS between two groups (73.1% [58.0–88.2%] vs 61.8% [52.2–71.4%], log-rank P = 0.21). Propensity-score matching further confirmed that adjuvant TKIs treatment extended the DFS (log-rank P = 0.024), but did not improve OS (log-rank P = 0.40). Our analysis revealed that adjuvant EGFR TKIs treatment was beneficial for early-stage NSCLC patients harboring activating EGFR mutations after complete resection.

## Introduction

Lung cancer is the most common malignancy, and is also the lead cause of cancer-related mortality worldwide^1^. Non–small-cell lung cancer (NSCLC) represents approximately 85% of all lung tumors, and adenocarcinoma is the most frequent histologic subtype of NSCLC^2^. The identification of subsets of lung cancer with oncogenic drivers has transformed the management of advanced NSCLC^3^. Activating mutations in the epithelial growth factor receptor (EGFR) are present in 10% to 15% of patients with lung adenocarcinoma in North America and up to 60% of patients in Asia^3, 4^. Small molecule tyrosine kinase inhibitors (TKIs) have achieved remarkable success in the treatment of advanced NSCLC harboring EGFR activating mutations^5–8^. EGFR TKIs has been recommended as first-line therapy for EGFR mutation-positive metastatic or recurrent NSCLC patients by major organization guidelines^9^.

On the constrast, little progress has been made in managing early-stage NSCLC recently, although about 10% to 65% of patient experienced fatal recurrence within five year after complete resection^10^. The meta-analysis by the Lung Adjuvant Cisplatin Evaluation (LACE) collaborative group demonstrated that adjuvant cisplatin-based chemotherapy significantly reduced disease recurrence and improved the survival of completely resected NSCLC patients^11^, and it has been recommended as routine clinical practice by major organizations.

Generally, drugs with the strongest activity are used in the adjuvant treatment for malignancies. An important example of using molecularly targeted agents in the adjuvant treatment for solid tumors is imatinib treatment for gastrointestinal stromal tumor (GIST)^12, 13^. Conversely, the addition of cetuximab, an EGFR antibody, to adjuvant chemotherapy failed to improve the survival of patients with KRAS wild-type resected stage III colon cancer^14, 15^. The efficacies of EGFR TKIs in the adjuvant treatment of NSCLC remain unclear. Randomized trials showed that adjuvant EGFR TKIs treatment did not prolong the survival of NSCLC patients after complete resection^16, 17^. Conversely, a single-arm phase 2 trials revealed that adjuvant erlotinib in resected EGFR mutation-positive NSCLC yielded excellent 2-year disease-free survival (DFS) (94%) compared to historical genotype-matched controls^18^. Currently, several large-scale clinical trials are ongoing to investigate the efficacies of adjuvant EGFR TKIs treatment in early-stage NSCLC, however, the final results wouldn’t be released until years later.

Herein, we performed a retrospective cohort analysis to test the hypothesis that adjuvant EGFR TKIs treatment could improve the survival of EGFR-mutant NSCLC patients receiving complete resection.

## Results

A total of 209 NSCLC patients harboring EGFR activating mutations were included in the study cohort. These patients all received complete resection, and were diagnosed with stage I to IIIA. Among them, 41 (19.6%) patients included EGFR TKIs in the adjuvant treatment regimen. The demographic and clinicopathologic characteristics of EGFR TKIs-treated group and the control group were summarized in Table 1. EGFR TKIs-treated group had higher proportion of elder patients than the control group (53.7% vs. 36.3%, P = 0.042). The distributions of gender, smoking status, pathologic stage, adjuvant chemotherapy were similar between the two groups.

**Table 1:** Demographic and clinicopathologic characteristics of EGFR-mutant NSCLC patients receiving complete resection.

Characteristic	Total (N = 209)		EGFR TKIs (N = 41)		Control (N = 168)		*P*
	No.	%	No.	%	No.	%	
Age, years							0.042
<60	126	60.3	19	46.3	107	63.7	
≥60	83	39.7	22	53.7	61	36.3	
Gender							0.11
Female	147	70.3	33	80.5	114	67.9	
Male	62	29.7	8	19.5	54	32.1	
Smoking status							0.93
Never	154	73.7	30	73.2	124	73.8	
Current/Former	55	26.3	11	26.8	44	26.2	
Stage							0.49
I	90	43.1	21	51.2	69	41.1	
II	68	32.5	11	26.8	57	33.9	
III	51	24.4	9	22.0	42	25.0	
Adjuvant chemotherapy							0.72
No	97	46.4	18	43.9	79	47.0	
Yes	112	53.6	23	56.1	89	53.0	

Abbreviation: EGFR TKI, epithelial growth factor receptor tyrosine kinase inhibitor.

### Survival and Cox regression analysis

**Figure 1:**
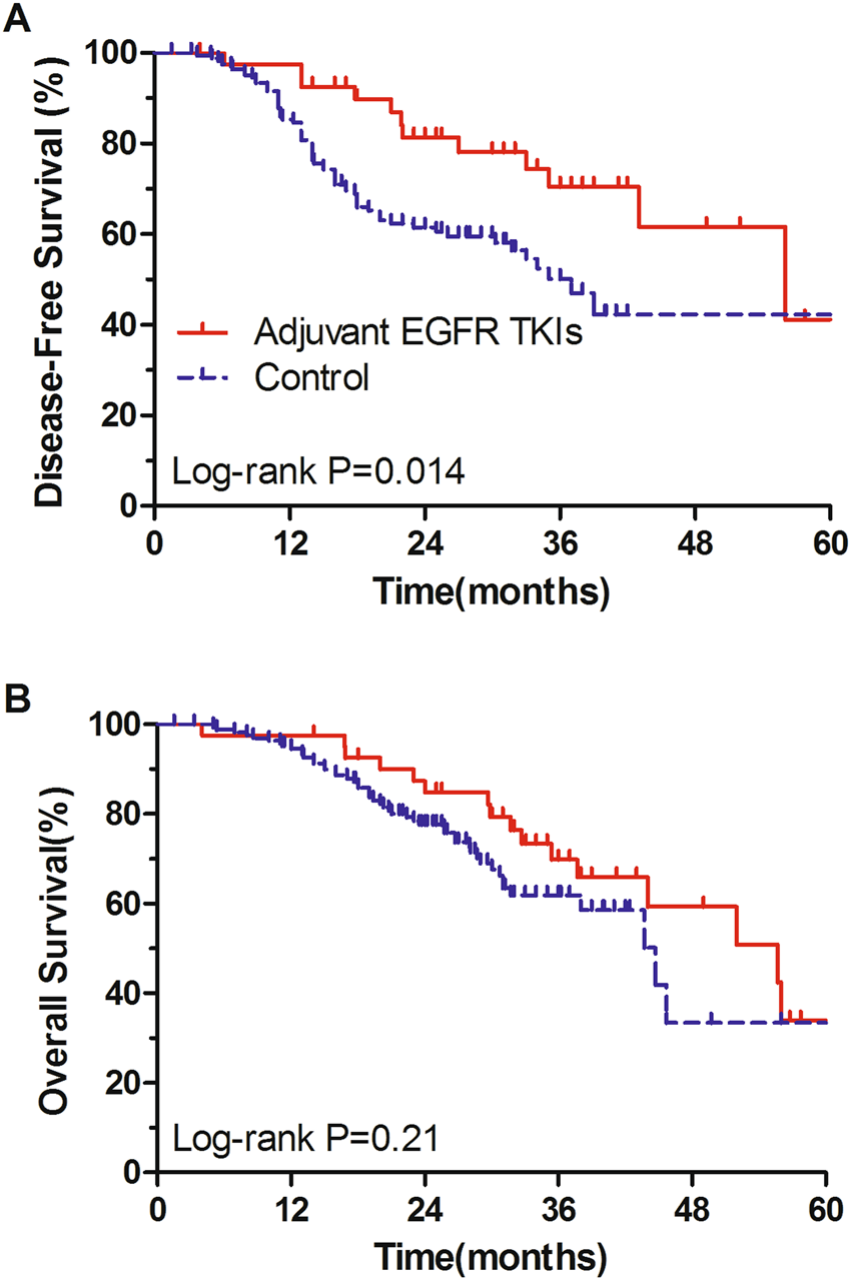
Disease-free survival (**A**) and overall survival (**B**) treated with or without EGFR TKIs after complete resection among all EGFR-mutant NSCLC patients.

The 3-year DFS of patients in the adjuvant EGFR TKIs treatment group and the control group were 70.5% (95% CI, 54.6–86.4%) and 50.2% (95% CI, 40–60.4%), respectively (Fig. 1A). Log-rank test showed that adjuvant EGFR TKIs treatment significantly prolonged DFS of EGFR-mutant patients (P = 0.014). On multivariate analysis, adjusted for age, gender, smoking status, pathologic stage and adjuvant chemotherapy, adjuvant EGFR TKIs treatment was significantly associated with improved DFS (HR, 0.51; 95% CI, 0.29–0.97; P = 0.04; Table 2).

**Table 2:** Multivariate analyses of DFS and OS in EGFR-mutant NSCLC patients receiving complete resection.

Resection type	DFS		OS	
	HR(95%CI)	*P*	HR(95%CI)	*P*
Adjuvant EGFR TKIs (Yes vs. No)	0.51(0.27–0.97)	0.04	0.78(0.44–1.41)	0.41
Pathologic stage		<0.001		<0.001
I	Reference		Reference	
II	1.64(0.89–3.02)	0.11	1.47(0.74–2.92)	0.27
III	3.04(1.72–5.37)	<0.001	3.63(1.95–6.75)	<0.001
Adjuvant chemotherapy (Yes vs. No)	0.67(0.43–1.06)	0.07	0.55(0.33–0.91)	0.02

Abbreviations: DFS, disease-free survival; OS, overall survival; HR, hazard ratio; 95% CI, 95% confidence interval.

The 3-year OS of patients in the adjuvant EGFR TKIs treatment group and the control group were 73.1% (95% CI, 58.0–88.2%) and 61.8% (95% CI, 52.2–71.4%), respectively (Fig. 1B). Log-rank test showed no significant difference in OS between the two comparison groups (P = 0.21). On multivariate Cox regression analysis, adjusted for age, gender, smoking status, pathologic stage and adjuvant chemotherapy, adjuvant EGFR TKIs treatment was not associated with OS (HR, 0.78; 95% CI, 0.44–1.41; P = 0.41; Table 2).

### Propensity-score matching analysis

**Figure 2:**
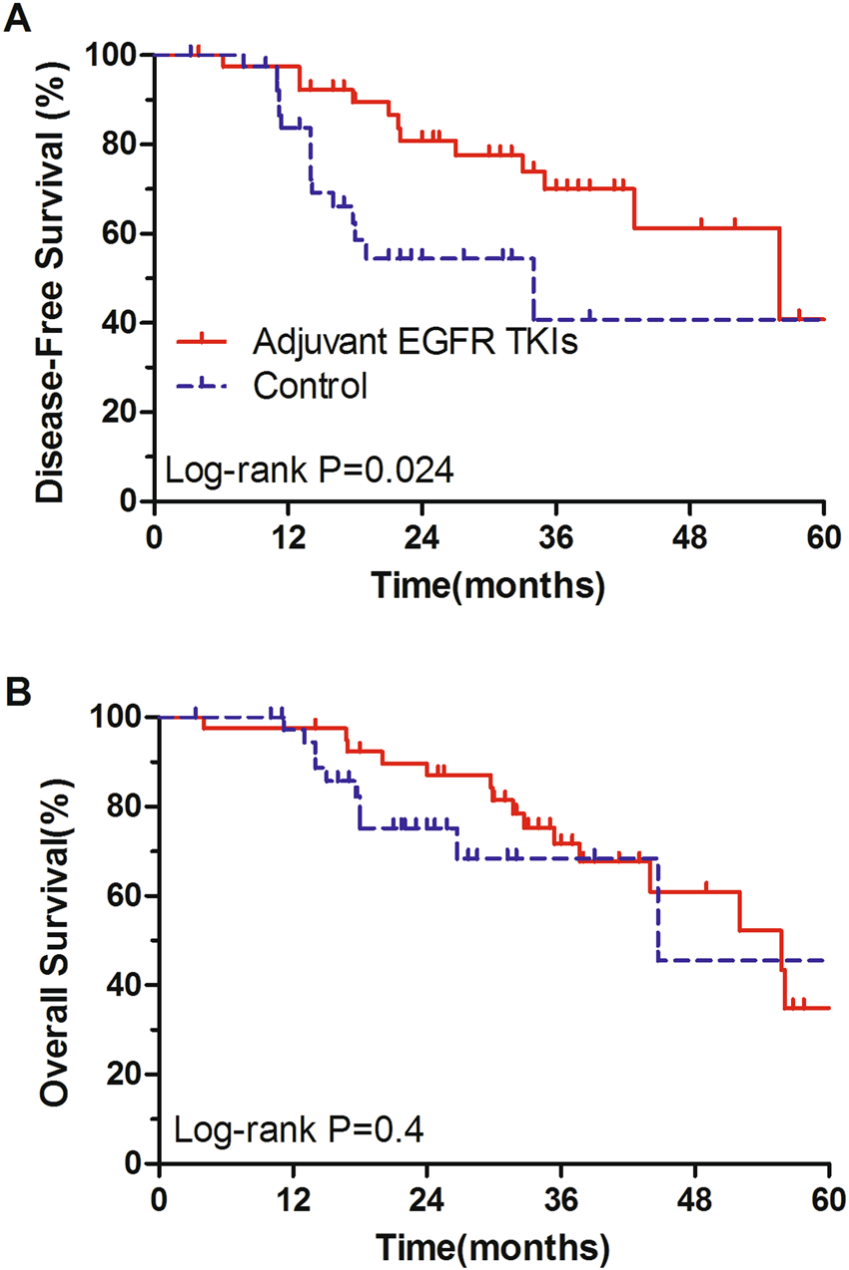
Disease-free survival (**A**) and overall survival (**B**) treated with or without EGFR TKIs after complete resection in the propensity-matched cohort of EGFR-mutant NSCLC patients.

To further investigate the efficacies of adjuvant EGFR TKIs treatment in EGFR-mutant NSCLC patients, propensity-score analysis was applied to match the patients in the two groups, using clinicopathologic covariates. The matching derived a cohort consisting of 40 pairs of patients, and the baseline clincopathologic characteristics were well-balanced between two groups. The DFS of patients receiving adjuvant EGFR TKIs treatment was significantly superior to that of control group (log-rank P = 0.024, Fig. 2A). There was no significant difference in OS between patients who received adjuvant EGFR TKIs treatment and those who did not (log-rank P = 0.40, Fig. 2B).

## Discussion

The current study retrospectively analyzed the clinical, treatment and survival data of 209 NSCLC patients harboring EGFR activating mutations who underwent complete resection of pulmonary tumors. The utility of EGFR TKIs in the adjuvant treatment significantly prolonged the DFS of these patients, and remained an independent predictor of favorable DFS on multivariate Cox regression analysis. Unfortunately, the benefit of DFS failed to convert to the benefit of OS. Propensity-score matching analysis was further employed to confirm the DFS benefit of adjuvant EGFR TKIs treatment for EGFR mutant patients.

Small molecular TKIs targeting EGFR activating mutations are the major breakthrough in the management of NSCLC during the past decades. For advanced NSCLC harboring EGFR activating mutations, EGFR TKIs treatment provided higher response rate (60–80%), and significantly extended progression-free survival (PFS)^5, 7, 19^. So far, first generation TKIs (erlotinib, gefitinib and icotinib), second generation TKIs (afatinib) and third generation TKIs (osimertinib) have already been approved for the treatment for advanced NSCLC harboring EGFR activating mutations.

Encouraged by the excellent efficacy of imatinib in the adjuvant treatment for GIST with KIT protein positive expression, oncologists seek to determine whether adjuvant EGFR TKIs treatment could improve the postoperative survival of NSCLC patients. It is until recent years that EGFR mutation status is demonstrated to be the strongest predictor for EGFR TKIs treatment effects. The first randomized clinical trials included all the patients with completely resected stage IB, II, and IIIA NSCLC^17^. Another trial included patients whose tumors were determined to be EGFR-positive by IHC and/or FISH (*EGFR* amplification or high polysomy)^16^. Both trials showed no survival benefit from adjuvant EGFR TKIs treatment over placebo. A phase II trial randomly assign patients with resected stage IIIA-N2 NSCLC harbouring EGFR mutations to receive pemetrex and carboplatin (PC) followed with or without gefitinib for 6 months, and found that DFS was significantly longer among those who received PC-gefitinib than those who received PC alone^20^. Therefore, the role of EGFR TKIs in the adjuvant treatment of NSCLC remains under great debate.

Our analysis revealed that adjuvant EGFR TKIs treatment significantly extended the DFS of patients harboring EGFR mutation, which was further confirmed by multivariate Cox regression analysis and propensity-score matching analysis, while EGFR TKIs treatment failed to improve the OS. These findings were in accordance with a recent meta-analysis by Huang *et al*.^21^. Several phase 3 trials, including ADJUVANT trial (NCT01405079), will determine the efficacy of EGFR TKIs in the adjuvant treatment for complete resected early-stage NSCLC harboring activating EGFR mutations in the near future.

A potential concern with adjuvant EGFR TKIs treatment is the early use of the inhibitors may change the biological behavior of tumors, and lead to the emergence of more resistant disease at the time of recurrence^22^. In the retrospective analysis of the phase 2 single-arm SELECT trial^23^, T790M mutation was detected in one patient among 15 patients with relapse who underwent repeat biopsy. The median PFS was 10 months, similar to that in the first-line setting. This suggests that EGFR TKIs retreatment for patients experiencing recurrence after adjuvant TKIs treatment remains feasible.

There are a few limitations that should be taken into consideration during data interpretation. First of all, the current study was potentially exposed to selection bias for the retrospective nature. Propensity-score matching was employed to compensate for some differences in baseline characteristics that may influence the outcomes, and further confirmed the efficacy of adjuvant EGFR TKIs treatment. Additionally, the duration of TKIs treatment was not collected and was inconsistent among the included patients. Indeed, the optimal TKIs treatment duration is still unclear for NSCLC in the adjuvant setting. The ongoing trial (NCT01746251) would be helpful to determine whether there is an optimal duration of adjuvant EGFR TKIs treatment.

In conclussion, the present propensity-matched analysis of NSCLC patients harboring EGFR activating mutations revealed that the utility of EGFR TKIs in the adjuvant treatment significantly improve the DFS after complete resection. EGFR TKIs could play an important role in the treatment for early-stage NSCLC.

## Patients and Methods

### Patients

All patients who underwent lung resection in our hospital from January 2010 to December 2013 were retrospectively analyzed. Medical records of patients were reviewed to collect information regarding clinicopathologic characteristics, treatment regimens and follow-up. The inclusion criteria were as follows: 1) histologically comfirmed NSCLC harboring activating EGFR mutations (Del 19 and L858R); 2) receiving complete resection; 3) pathologic stage I to IIIA; 4) postoperative treatment regimens and follow-up available. EGFR mutations detection applied direct sequencing before June 2013, and shifted to Amplified Refractory Mutation System (ARMS) method afterwards according to the institutional protocol. After selection, a total of 209 patients were eligible for our analysis. Patients receiving EGFR TKIs, including erlotinib, gefitinib and icotinib, as adjuvant treatment were classified as adjuvant EGFR TKIs treatment groups, and the rest were control group. This study was performed in line with the Helsinki Declaration and approved by the Institutional Review Board (IRB) of the Affiliated Hospital of Qingdao University. Written informed consent was waived by the IRB for its retrospective nature. The experiment protocol of this study was strictly conducted in accordance with the guidelines.

The preoperative workup routinely included chest and upper abdomen computed tomographic (CT) scans, bronchoscopic examination with biopsy when possible, and CT scanning or magnetic resonance imaging (MRI) of the brain. Nuclear medicine bone scan was performed when clinically indicated. All patients underwent complete resection of pulmonary tumors and systematic mediastinal lymph node dissection or sampling through thoractomy or video-assisted thoracic surgery. Written informed consent consent was obtained from all patients before proposed surgical resection. All resected specimens were fixed in 10% formalin and stained with hematoxylin and eosin, and the pathological slides were evaluated by experienced pathologists. Tumors were staged according to the 7th edition of the American Joint Committee on Cancer (AJCC) - Union for International Cancer Control (UICC) staging system for cancer^24^.

### Outcomes and follow-up

The primary endpoint was DFS, which was defined as the time interval between the date of pulmonary resection and the date of either first recurrence of cancer or the last follow-up. Recurrent disease (either local or distant) was histologically confirmed whenever possible. Lung recurrence and second primary lung cancer were differentiated according to the method proposed by Girard *et al*.^25^. The secondary endpoint was overall survival (OS), which was defined as the time interval between the date of pulmonary resection and the date of either death of any cause or the last follow-up.

The postoperative surveillance protocol was in accordance with National Comprehensive Cancer Network guidelines^9^. Patients were recommended to come to outpatient department for surveillance every 3–6 months for the first two years, and then annually for the subsequent years. Patients received a physical examination, and chest and upper abdominal CT scans. Brain MRI or CT scan and PET scan were performed when clinically indicated. The follow-up was performed by the outpatient clinic or official contact with patients or their relatives by telephone. The last follow-up was 30th June 2016. The median follow-up was 49.3 months.

### Statistical analysis

Patients’ baseline clinical and pathologic parameters were recorded as categorical variables, and their distributions between treatment and control groups were compared by χ2 test. Survival curves of DFS and OS were plotted by using Kaplan-Meier method and compared by using the log-rank test. All parameters which attained a significance level of p < 0.10 in a univariate analysis were entered into the multivariate Cox proportional hazard regression analysis to calculate the hazard ratios (HR) and 95% confidence intervals (CI). A backward stepwise regression procedure was applied.

Because of the retrospective nature of the current study exposing to potential selection bias, we conducted a propensity score matching analysis to address the imbalance in some baseline characteristics between the two groups^26^. The propensity score, modeling the probability that a patient is assigned to the EGFR TKIs treatment or control group as a consequence of the individual profile of these factors in a nonrandomized patient, was calculated from a logistic regression model, based on the baseline clinicopathologic parameters. Then, a one-to-one match without replacement was performed by using nearest neighbor matching method with a caliper of 0.1, to form matched pairs, leading to an even distribution of potential confounding factors between the two groups. All statistical analyses were carried out using SPSS 22.0 software for Windows (SPSS Inc., Chicago, IL). Statistical significance was set at P < 0.05 and all tests were two sided.
